# Preliminary estimation of the prevalence of chemotherapy-induced dysgeusia in Japanese patients with cancer

**DOI:** 10.1186/1472-684X-12-38

**Published:** 2013-10-29

**Authors:** Hiroo Imai, Hiroshi Soeda, Keigo Komine, Kazunori Otsuka, Hiroyuki Shibata

**Affiliations:** 1 Department of Clinical Oncology, Graduate School of Medicine, Akita University, Hodo 1-1-1, Akita, Akita, Japan

**Keywords:** Taste alteration, Dysgeusia, Quality of life, Oral 5-fluorouracil analogs, Paper test strip

## Abstract

**Background:**

Although dysgeusia is a common adverse event in chemotherapy patients; it has not been evaluated using objective methods, and its prevalence and frequency have not been quantified.

**Methods:**

Salt-impregnated taste strips were used to objectively assess dysgeusia in patients receiving chemotherapy at Akita University (n = 38) and those off chemotherapy (n = 9). Participant characteristics, and ongoing and previous chemotherapies were evaluated, and their associations with dysgeusia analyzed.

**Results:**

Dysgeusia developed in 38.8% (14/38) of chemotherapy patients, and was most prevalent in patients receiving 5-fluorouracil (5-FU) or its oral analogs (48.1%, 13/27). Particularly, dysgeusia developed in 55.6% (10/18) of patients receiving oral 5-FU analogs; however, prevalence in patients receiving and off chemotherapy was not significantly different. Patients aged ≥70 years also tended to experience dysgeusia (75.0%, 6/8).

**Conclusions:**

Association with dysgeusia may be higher for some chemotherapeutic drugs. Dysgeusia should be routinely assessed in chemotherapy patients with objective methods such as paper strips; interventions for its prevention may be required.

## Background

Maintenance of quality of life (QOL) during chemotherapy is important for patients with cancer. Although alteration of taste (dysgeusia) is a nonlethal condition that is often ignored, taste is critical to the pleasure of eating, which is a major part of QOL [1,2]. Dysgeusia is frequent in patients with cancer [3,4]. Care should be taken to prevent dysgeusia, and when identified, appropriate therapy should be provided. Research has shown that 60% of patients with advanced cancer experience dysgeusia even without anticancer therapy [5].

Pathophysiological mechanisms of dysgeusia during chemotherapy are explained by factors such as neurological damage in cranial nerves (VII, IX, and X) and taste buds and mucosal damage [6]. Dysgeusia in the cancer patient population is difficult to assess, and a quantitative, validated methodology for evaluation has not yet been established. Objective clinical research has not been adequate and, often relies on anecdotal information. In this study, a salt-impregnated taste strip was used to evaluate dysgeusia because of its objectiveness, ease, and low cost. As reported in the literature, a number of chemotherapeutic drugs, including cisplatin, doxorubicin, 5-fluorouracil (5-FU), docetaxel, and paclitaxel, can induce dysgeusia [7]. These drugs, along with the new oral 5-FU analogs, capecitabine and S1, are used in the management of various types of malignancies [8-13]. Because reports on the effects of these therapies on taste have been anecdotal, we used an observational objective approach to document and assess the actual prevalence of dysgeusia in patient groups receiving chemotherapy and those off chemotherapy (on- and off-chemotherapy groups, respectively).

## Methods

Patients of the Department of Clinical Oncology, Akita University Hospital between February 2012 and December 2012 participated in this cohort study.

To objectively evaluate dysgeusia, we used the Salsave kit® (AdvanTec, Tokyo, Japan), which is a validated salt test using paper strips with 6 concentrations of sodium chloride: 0.6%, 0.8%, 1.0%, 1.2%, 1.4%, and 1.6%. Participants initially tasted a paper strip with no salt crystals. The test was then readministered step-by-step using strips with lower to higher salt concentrations. Between each step, each participant’s mouth was cleansed with distilled water before the next tasting. The threshold of the recognized concentration was recorded as a grade of dysgeusia. If the participant could not recognize a 0.6% salt concentration, we deemed the participant’s taste perception to have been altered. Dysgeusia was recorded with each therapy for each individual.

The Stat Mate III (ATMS, Tokyo, Japan) was used to calculate relative risks. The level of statistical significance was set to *P* < 0.05. This study was scientifically and ethically approved by the Ethics Committee of the School of Medicine of Akita University (#791). Written informed consent was obtained from each patient for participation and publication of this cohort study.

## Results

### Participants

The participants included 31 male and 15 female patients, with an age range of 36–85 years and a median age of 64 years. All participants were diagnosed with stage IV malignancies (Table 1). Nine patients did not undergo chemotherapy while the study was being conducted; corresponding chemotherapies administered to the other 38 participants are listed in Table 1. Patients receiving 5-FU-based therapy were most frequently enrolled (27/38, 71.1%), followed by those receiving platinum (Pt)-based therapy (16/38, 42.1%) and those receiving taxane (Tx)-based therapy (5/38, 13.2%). Other comorbidities known to induce dysgeusia, such as diabetes mellitus (DM) [14], brain disease (BD) [15], and history of head and neck irradiation, were also evaluated [16]. Notably, of the patients who had not received chemotherapy, 1 had DM (11.1%, 1/9) and another had BD (11.1%, 1/9); whereas of patients who received chemotherapy, 3 had DM (7.9%, 3/38) and 6 had BD (15.8%, 6/38). During this study, 16 patients (42.1%, 16/38) received ≥ 2 chemotherapies; no patient received head and neck irradiation, but 1 underwent surgical resection for laryngeal sarcoma. However, no patient suffered from oral mucositis or candidiasis in any of the treatment groups.

**Table 1:** Background of participants

**Participants**	**On-chemotherapy**		**Off-chemotherapy**	
	**(n = 38)**		**(n = 9)**	
Age	36–85		54–73	
	(median, 64)		(median, 63)	
Gender	Male	25	Male	6
	Female	13	Female	3
Malignancy	Colorectal	14	Colorectal	4
	Gastric	12	Gastric	2
	Esophageal	5	STS	1
	NET	3	Melanoma	1
	STS	2	Bile duct	1
	Melanoma	2		
	Bile duct	0		
Chemotherapy	5-FU-based	27		
	Pt-based	16		
	Tx-based	5		
	Others	5		

*NET* neuroendocrine tumor, *Pt* platinum, *STS* soft tissue sarcoma, *Tx* taxane.

#### Prevalence of dysgeusia during chemotherapy

Of the 9 patients who did not receive chemotherapy (aged 54, 57, 57, 61, 63, 67, 70, 71 and 73 years; median age, 63 years), 7 successfully recognized the minimal salt concentration (0.6%) and 2 did not (22.2%, 2/9; Table 2). Even patients in the off-chemotherapy group experienced altered taste because of their past histories of chemotherapies or other comorbidities. Of this group, 1 patient had received chemotherapy with S1 more than a year previously. The other was chemotherapy-naïve but had a history of BD. Among the other 7 patients with no signs of dysgeusia, none had DM, although 1 experienced liver cirrhosis. Of the 38 on-chemotherapy group patients, 14 (36.8%; age: 36–85 years; median age: 64 years) could not recognize the minimal level (Table 2); among these 14 patients, 5 (35.7%) experienced comorbidities (cerebrovascular disease, epilepsy, brain metastasis, resected laryngeal sarcoma, and DM). Among the 24 patients who could recognize the minimal level, 4 (16.7%) experienced comorbidities (cerebrovascular disease, hypothalamic adenoma, meningioma, and DM); 1 patient had both BD and DM. Therefore, patients with comorbidities appeared to experience dysgeusia at a rate that was 1.79 times higher than that experienced by patients with no comorbidity [not significant (NS)]. The relative risk of dysgeusia between the on- and off-chemotherapy groups was 1.66 (NS).

**Table 2:** Dysgeusia experienced in each patient

**Age**	**≤59**	**60–69**	**70–79**	**≥80**	**Total**
Therapy					
5-FU total	4/6 (66.7)	2/10 (20.0)	4/8 (50.0)	3/3 (100)	13/27 (48.1)
Oral analogs	2/3 (66.7)	2/7 (28.6)	4/6 (66.7)	2/2 (100)	10/18 (55.6)
5-FU	2/3 (66.7)	0/3 (0)	0/2 (0)	1/1 (100)	3/9 (33.3)
Pt	2/3 (66.7)	2/9 (22.2)	0/4 (0)	-	16/38 (42.1)
Tx	1/3 (33.3)	0/1 (0)	1/1 (100)	-	5/38 (13.2)
Others	0/3 (0)	1/6 (16.7)	0/1 (0)	-	1/10 (10.0)
Off	0/2 (0)	1/3 (33.3)	1/3 (33.3)	-	2/8 (25.0)

The number of patients who experienced dysgeusia during therapy is indicated per total number undergoing chemotherapy. Numbers in parentheses indicate the percentage. Type of chemotherapy is indicated in the first column. *Off* off-chemotherapy.

Subgroup analysis was performed, although categorizing the on-chemotherapy group patients on the basis of specific drugs was difficult because of overlapping combination therapies. Dysgeusia was experienced in 48.1% (13/27) patients who received 5-FU, 25.0% (4/16) of those receiving Pt, 40.0% (2/5) of those receiving Tx, and 10.0% (1/10) of those receiving other therapies (Table 2). Compared with the off-chemotherapy group, the relative risk of dysgeusia was 2.2 for the oral 5-FU analog therapy (NS), 1.3 for the intravenous 5-FU therapy (NS), 1.0 for the Pt group, 1.6 for the Tx group (NS), and 0.4 for patients receiving other therapies. Although the relative risk for oral 5-FU analogs was not significant, it was higher than the relative risk for the other therapies. These data suggest that among frequently used chemotherapeutic drugs, oral 5-FU analogs could be a major risk factor for dysgeusia. Therefore, in this study, subsequent analyses primarily focused on oral 5-FU-based chemotherapy [17]. Among the 18 patients who received oral 5-FU analogs, the prevalence of dysgeusia was as high as 75.0% (6/8) in the patients aged ≥ 70 years, whereas it was 40.0% (4/10) in those aged < 70 years (Table 2). Compared with the patients aged < 70 years (NS), the relative risk of dysgeusia in the patients aged ≥ 70 years was 1.8 which implies that oral 5-FU-based therapies cause dysgeusia at a much higher frequency in patients aged ≥ 70 years than in those aged < 70 years.

### Grades of dysgeusia

**Figure 1:**
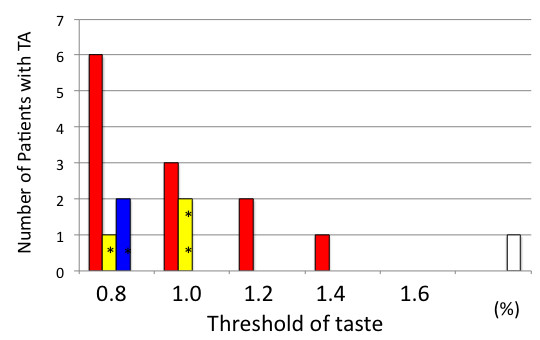
**Range of the worst values of taste recognition recorded in each patient**. The bars indicate the patients who received 5-fluorouracil (5-FU)-based (red), platinum (Pt)-based (yellow), taxane (Tx)-based (blue), and ifosfamide-based (white) therapies. *indicates combination therapy with 5-FU.

Among patients with dysgeusia, the median score was 0.9% (range: 0.8–1.6%). The most severe case was seen in a patient with local recurrence of laryngeal sarcoma who was receiving ifosfamide; he could not recognize a 1.6% salt concentration. Further, 50% patients affected by dysgeusia could not recognize a 0.8% salt concentration. The 13 patients with dysgeusia who received 5-FU-based therapy had a median score of 0.9% (range: 0.8–1.4%; Figure 1). All 4 of those with dysgeusia who received Pt-based therapies combined them with 5-FU therapies; the median for this group was 1.0%, (range: 0.8–1.0%; Figure 1). The two patients with dysgeusia who received Tx (one in combination with 5-FU) had scores of 0.8% (Figure 1). Notably, the severity of dysgeusia tended to worsen in patients who received 5-FU-based therapy.

### Reversibility of chemotherapy-induced dysgeusia

**Figure 2:**
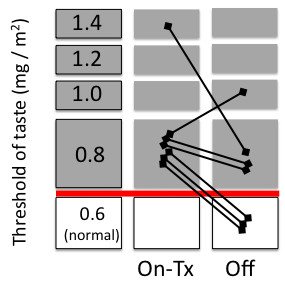
**Reversibility of dysgeusia**. The change in dysgeusia in each patient is plotted both on-chemotherapy and just before the next therapy cycle (off).

We attempted to assess whether the taste function could recover after chemotherapy in this study group. However, we could not document the experience of all patients during the entire chemotherapy period. Eight patients who received 5-FU-based therapy agreed to multiple assessments of dysgeusia several times during the course of their chemotherapy. Among these, recovery of normal taste function just before the start of the next cycle was confirmed in only 3 patients (ages 53, 75, and 77 years; Figure 2). Recovery of normal taste sensation could not be confirmed in 5 patients (ages 53, 67, 68, 75 and 78 years; Figure 2); furthermore, 4 of these 5 patients had a prior history of chemotherapy, which may have had an impact on the taste sensations.

## Discussion

The prevalence of dysgeusia due to recent chemotherapy was 38.8% in our hospital. Objective evaluation of the impact of dysgeusia in the clinic requires several types of procedure, including electrogustometry, whole-mouth gustatory testing, and magnetoencephalography [18-20]. However, because these procedures are labor-intensive, they are not suitable for routine evaluation. Among patients with phantogeusia and parageusia, in one study, 38% reported salty and 22% reported mixed sensations, such as bitter–salty or sour–sweet [21]. Evaluation of all 5 tastes, including salty, sweet, bitter, sour, and umami is labor-intensive. Reportedly, except for umami, these tastes are sensitive to radiotherapy to the same extent [16]. Therefore, alternative approaches are warranted. In this study, we used the Salsave kit® paper testing because of its ease of use during routine examinations [22]; it can easily diagnose dysgeusia, and is adequate for mass screening and follow-up tests. In addition, this taste strip could objectively estimate the grade of dysgeusia. Our study indicated that oral 5-FU analogs may induce dysgeusia during therapy. These drugs are important therapies for various types of malignancies, including colorectal, gastric, mammary, and pulmonary cancers, which are the most common cancers worldwide. Therefore, in any large-scale study of dysgeusia in patients receiving oral 5-FU analogs, the paper strip test would be convenient for assessing a larger number of participants.

Interventions to decrease dysgeusia should be developed to support QOL of patients who receive chemotherapy. Zinc consumption has been reported to improve taste sensation affected by radiation [23]. Although glutamine is known to ameliorate neuropathy induced by cisplatin and paclitaxel in rats [24], a phase III trial using oral glutamine failed to prove that it could prevent dysgeusia caused by taxanes [25]. A standard therapy for dysgeusia caused by cancer and cancer therapy has not yet been established [26]. Although the importance of supportive care during chemotherapy, including use of antiemetics, has been recognized [27,28], future clinical studies in this regard are warranted. Our study was observational, and we did not detect any statistically significant differences in the prevalence of dysgeusia between the on- and off-chemotherapy group patients; this could be attributable to the limited sample size.

## Conclusions

We demonstrated that the prevalence of dysgeusia can be routinely assessed by using an objective, easy-to-use, and low-cost method involving paper strip testing in patients who receive chemotherapy. Many patients with cancer experience dysgeusia caused by chemotherapy. Although our results should be confirmed by further investigations in a larger patient population, our findings suggest that potential dysgeusia in cancer patients should be addressed to protect their QOL.

## Abbreviations

5-FU: 5-fluorouracil; BD: Brain disease; DM: Diabetes mellitus; NS: Not significant; QOL: Quality of life.

## Competing interests

The authors declare that they have no competing interests.

## Authors’ contributions

HI, HSo, KK, and KO acquired and analyzed the data. HSh planned and interpreted the data. All authors read and approved the final manuscript.

## Authors’ information

HI, HSo, KK, and KO are assistant professors; KO is a lecturer; HSh is a professor in the Department of Clinical Oncology. All authors, except KK, are certified as specialists in medical oncology by the Japanese Society of Medical Oncology.

## Pre-publication history

The pre-publication history for this paper can be accessed here:

http://www.biomedcentral.com/1472-684X/12/38/prepub
